# Optimization of spectral photon-counting CT reconstruction parameters for improved detection of peritoneal metastases compared with energy-integrating detector dual-energy CT: a phantom study

**DOI:** 10.1186/s41747-026-00816-w

**Published:** 2026-09-30

**Authors:** Joël Greffier, Rémi Grange, Djamel Dabli, Angèle Houmeau, Maria Nicole Antonuccio, Pascal Rousset, Salim Si-Mohamed

**Affiliations:** 1 IMAGINE UR UM 103, Montpellier University, Department of Medical Imaging, Nîmes University Hospital, Nîmes, France; 2 Department of Radiology, University Hospital of Saint-Etienne, Saint-Priest-en-Jarez, France; 3 CICLY, EMR 3738, Lyon 1 University, Lyon, France; 4 Université de Lyon, INSA-Lyon, Université Claude Bernard Lyon 1, UJM-Saint Etienne, CNRS, INSERM, CREATIS UMR 5220, Villeurbanne, France; 5 CT Clinical Science, Philips, Suresnes, France; 6 Department of Radiology, CHU Lyon-Sud, Hospices Civils de Lyon, Pierre-Bénite, France; 7 Department of Radiology, Hôpital Louis Pradel, Hospices Civils de Lyon, Bron, France

**Keywords:** Colorectal cancer, Dual-energy, Peritoneal metastases, Photon-counting CT, Task-based image quality assessment

## Abstract

**Objective:**

To determine the best combination of reconstruction parameters on a spectral photon-counting CT (SPCCT) system to improve detection of peritoneal metastases (PMs) on virtual monoenergetic images (VMIs) at 40 keV.

**Materials and methods:**

A spectral phantom was scanned using SPCCT and dual-energy CT (EID-DECT) at 8.5 mGy (CTDI_vol_). 40 keV VMIs were reconstructed on EID-DECT using a level 4 Spectral algorithm and kernel B. For SPCCT, two reconstruction kernels (STD-B and HRB) and four iDose^4^ levels (i5/i7/i9/i11) were used. Noise power spectrum (NPS) and task-based transfer function (TTF) were analyzed. Detectability indices (*d*’) for non-mucinous and mucinous peritoneal metastases (nmPMs and mPMs) were calculated.

**Results:**

Noise magnitude (-22.4 ± 0.8%) and noise texture (*f*_av_) values were lower (-21.3 ± 4.4%), but spatial resolution (*f*_50_) was higher (5.8 ± 3.4%) with STD-B than with HRB at all iDose^4^ levels. For both simulated PMs, *d*’ values were higher with STD-B than with HRB (22.3 ± 0.3%). Compared to EID-DECT, noise magnitude and *d*’ values were similar or better with STD-B at i7/i9/i11 and i9/i11 for HRB. At all iDose^4^ levels and with both kernels, *f*_av_ or *f*_50_ values were higher with SPCCT, except for *f*_50_ at i11 with HRB.

**Conclusion:**

Optimized reconstruction settings on SPCCT offer better image quality and lesion detectability on 40 keV VMIs than EID-DECT. Combining the STD-B kernel with level i9 achieved the best compromise between noise texture, spatial resolution, and detectability for simulated PMs.

**Key Points:**

***Question*** Can the performance of a spectral photon-counting CT (SPCCT) improve detection of peritoneal metastases (PM on 40 keV images compared with a conventional dual-energy CT (EID-DECT) system?

***Findings*** SPCCT with optimized reconstruction settings improved image noise, spatial resolution, and lesion detectability compared with EID-DECT at 40 keV.

***Relevance Statement*** SPCCT may enhance the preoperative assessment of colorectal cancer patients by better visualizing small or low-contrast peritoneal lesions. Validation in larger clinical cohorts is required to confirm these findings and define standardized reconstruction protocols.

**Graphical Abstract:**

**Figure:**
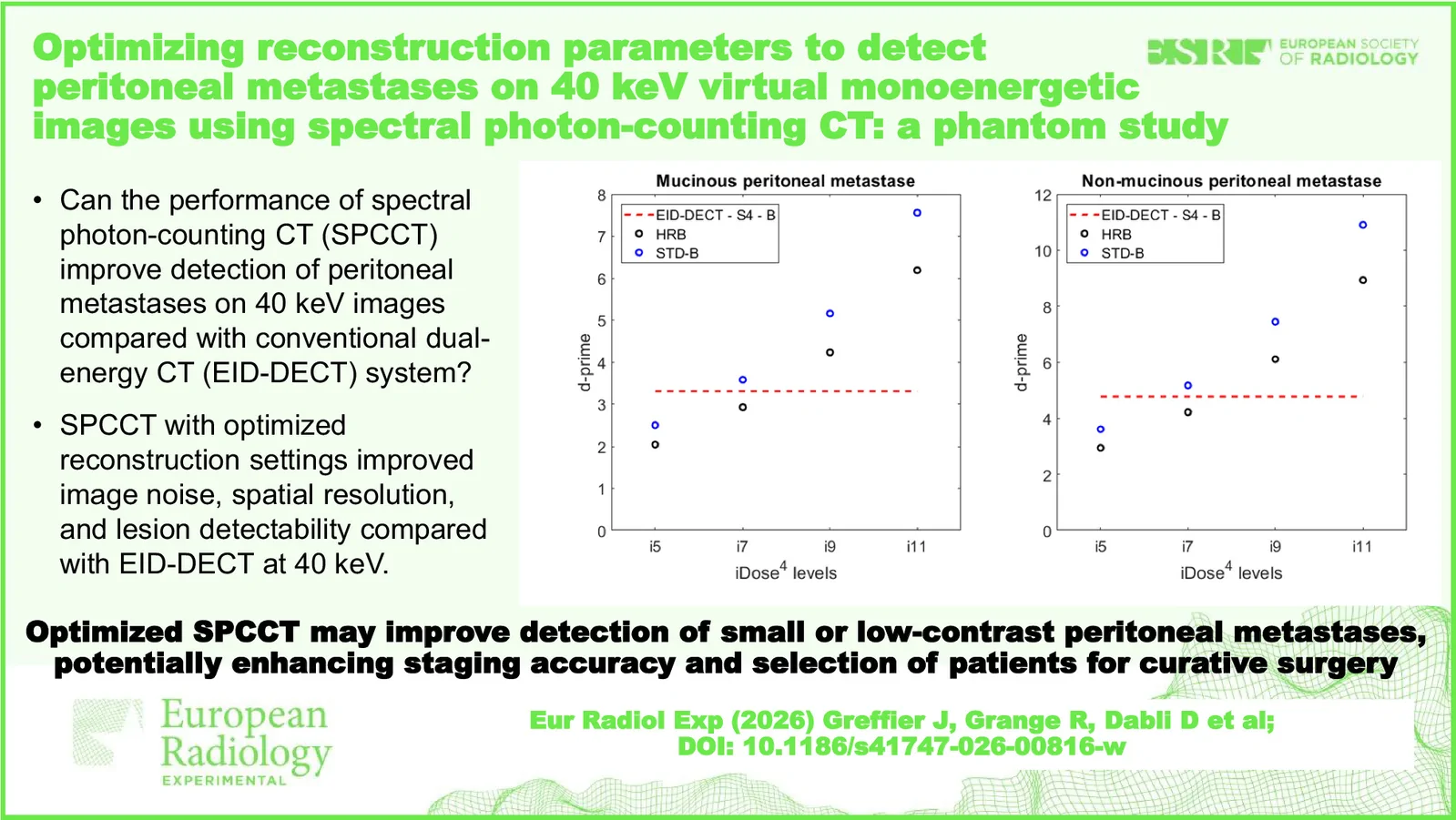

## Background

Colorectal cancer is one of the most common cancers worldwide and a major cause of cancer-related mortality, with approximately half of patients presenting with metastases at diagnosis. Peritoneal metastases (PMs) are associated with the poorest prognosis among metastatic sites. However, early detection may improve patient management, facilitate curative surgery, and ultimately improve tumor control, survival outcomes, and quality of life [1, 2]. In oncology, computed tomography (CT) is the imaging modality of choice in current practice, playing a key role in detecting these lesions, selecting patients for surgery, and avoiding unnecessary procedures [3]. However, CT clearly underestimates PMs as it is limited by its low soft-tissue contrast, often leading to an underestimation of the peritoneal disease burden, particularly when the lesions are small [4, 5]. Sensitivity mainly depends on nodule size, with several studies reporting low sensitivity of approximately 11% for nodules < 5 mm, 25–50% for lesions < 1 cm, and excellent sensitivity for lesions > 5 cm [6, 7].

Dual-energy CT (DECT), which involves acquiring or detecting two photon spectra at low and high energies, generates spectral images that offer promising improvements in contrast and lesion detectability. One recent study demonstrated that virtual monoenergetic images (VMIs) at 40 keV improved the contrast-to-noise ratio of PMs, particularly non-mucinous PMs, paving the way to integrate spectral imaging into preoperative CT protocols for colorectal cancer patients with suspected PMs [4]. However, 40 keV VMIs generated on the DECT platforms currently used in clinical practice have limitations, including greater image noise, lower spatial resolution, and degraded noise texture (*e.g*., image smoothing) [8, 9].

Recently, photon-counting CT systems based on photon-counting detectors (PCDs) have been developed, including the clinical spectral photon-counting CT prototype (SPCCT) developed by Philips Healthcare. Compared with the energy-integrating detectors (EIDs) used in conventional CT and DECT systems, PCDs eliminate electronic noise, improve spatial resolution, and provide greater dose efficiency [10, 11]. In addition, PCDs detect and quantify individual X-ray photons according to their energy, offering refined spectral sampling across multiple energy bins. This technology allows more accurate characterization of photoelectric and Compton interactions and generates higher-quality spectral images, particularly low-energy VMIs. Taken together, these advantages may make it possible to generate less noisy, higher-resolution VMIs at 40 keV, potentially improving the detectability of PMs [12]. However, the clinical performance of SPCCT depends not only on detector technology but also on image reconstruction. Reconstruction parameters, including reconstruction kernels and iterative reconstruction levels, substantially influence image noise, noise texture, spatial resolution, and lesion detectability. Identifying the optimal combination of reconstruction parameters is therefore essential to fully exploit the potential of SPCCT in clinical practice. Nevertheless, as this technology remains relatively recent, no studies have compared the performance of SPCCT and EID-DECT systems for detecting PMs. Furthermore, multiple reconstruction parameter combinations are available on SPCCT systems, and a comprehensive evaluation of their impact on noise magnitude, noise texture, spatial resolution, and lesion detectability is required before clinical implementation.

The purpose of this preliminary phantom study was to find the best combination of reconstruction parameters on a SPCCT system to improve the detection of PMs on 40 keV VMIs. A task-based image quality assessment was performed to this end.

## Materials and methods

### Phantom

**Fig. 1:**
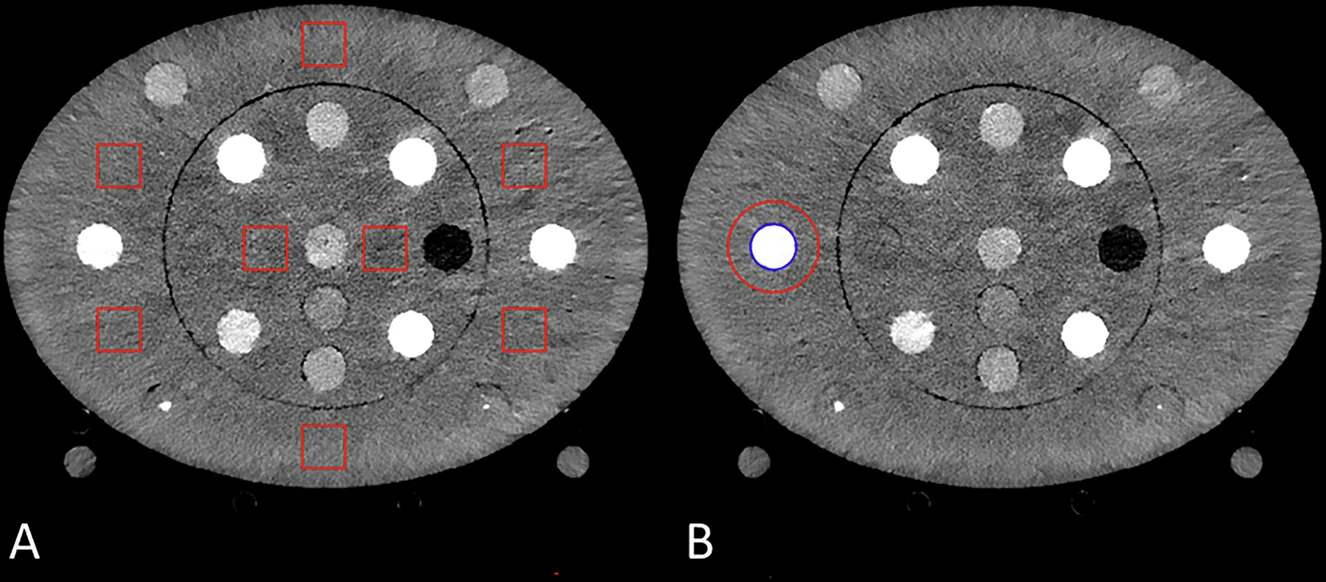
CT images of the phantom used in this study and the obtained energy-integrating DECT (WL: 60 HU and WW: 360 HU). **A** Eight square regions of interest (ROIs) of 32 × 32 pixels were used to evaluate the noise power spectrum (NPS) on VMIs at 40 keV. **B** Circular region of interest used to compute the task-based TTF on an insert of blood-mimicking material plus iodine at 2 mg/mL on VMIs at 40 keV

Acquisitions were made on the elliptical Multi-Energy CT phantom (Gammex, Sun Nuclear), equipped with 16 removable inserts (2.8 cm in diameter) embedded in a water-equivalent background material. All inserts were positioned identically for each CT acquisition (Fig. 1). The blood-mimicking material with iodine at 2 mg/mL insert (BMMI2) was used to calculate the task-based transfer function (TTF) and to simulate the detectability of non-mucinous and mucinous PMs during the portal phase This insert was chosen because its theoretical contrast at 40 keV is similar to that of both lesions (232 Hounsfield unit (HU) *versus* 290 and 201 HU, respectively). It was placed in a lateral position within the phantom to calculate the TTF.

## Acquisition and reconstruction parameters

Acquisitions were made on a Spectral CT7500 dual-layer CT system (Philips Healthcare) equipped with energy-integrating detectors (EID-DECT) and on the advanced research prototype SPCCT system (Philips Healthcare) equipped with cadmium–zinc–telluride-based photon-counting detectors [13, 14]. The acquisition parameters were close to those proposed by R. Grange et al. for detecting PMs [4]. Ten acquisitions were made on each CT system with a tube voltage of 120 kVp, a rotation time of 0.5 s/rotation, a pitch factor of 1.241, and a fixed tube current of 273 mA (tube current modulation was disabled). The physical beam collimation was 128 × 0.625 mm for EID-DECT and 64 × 0.275 mm for SPCCT, and the volume CT dose index (CTDI_vol_) was close to 8.5 mGy for both systems.

As recommended by R. Grange et al [4], VMIs at 40 keV were reconstructed with a 420 mm field of view, a 512 × 512 pixel matrix size, and a slice thickness of 1.5 mm (1.5 mm increment). For EID-DECT, VMIs were reconstructed using level 4 of the hybrid iterative reconstruction algorithm, “Spectral”, and with the standard soft tissue kernel “B”. These settings corresponded to those routinely used in clinical practice at our institutions for abdominal and pelvic spectral imaging. To determine the best combination of reconstruction parameters for SPCCT, VMIs were reconstructed using levels 5, 7, 9, and 11 of the hybrid iterative reconstruction algorithm “iDose⁴” with the “STD-B” and “HRB” kernels. These iDose⁴ levels were selected to span the range from the lowest level providing acceptable image noise (i5) to the highest available level (i11). The “STD-B” and “HRB” kernels were recommended by Philips Healthcare and were considered the most appropriate for abdominal and pelvic spectral imaging with SPCCT.

## Task-based image quality assessment

A task-based image quality assessment was performed on VMIs using iQMetrix-CT v1.2 software [15] following the same methodology as previously published [8, 16, 17]. To limit the noise effect on the Edge Spread Function curves used to compute the TTF curves [18], TTF and therefore NPS were calculated for all images from 10 acquisitions, rather than individually for each acquisition.

### Noise power spectrum (NPS)

The NPS was computed in 200 consecutive axial slices (20 slices for each of the 10 acquisitions) by placing eight square regions of interest (ROIs) of 32 × 32 pixels (Fig. 1A) according to the following equation (Eq. 1):

1 $${{NPS}}_{2D}\left({f}_{x},{f}_{y}\right)=\frac{{\Delta }_{x}{\Delta }_{y}}{{L}_{x}{L}_{y}}\frac{1}{{N}_{{ROI}}} {\sum}_{i=1}^{{N}_{{ROI}}}{|{{FFT}}_{2D}\left\{{{ROI}}_{i}\left(x,y\right)-{{FIT}}_{i}(x,y)\right\}|}^{2}$$

where in Δ_*x*_ and Δ_*y*_ are the pixel size in the *x*- and *y*-directions (mm), respectively; FFT is the Fast Fourier Transform; *L*_*x*_ and *L*_*y*_ are the lengths of the ROIs in pixels in the *x*- and *y*-directions; *N*_ROI_ is the number of ROIs; ${{ROI}}_{i}\left(x,y\right)$ is the pixel value for the ith ROI at position (*x*, *y*) and ${{FIT}}_{i}(x,y)$ is a 2nd order polynomial fit of ${{ROI}}_{i}\left(x,y\right)$.

To quantify changes in noise magnitude, the square root of the area under the NPS2D curve was measured. The average spatial frequency (*f*_av_, in mm^−1^) of the NPS1D curve was also measured to quantify changes in noise texture [18].

### Task-based TTF

TTF was computed from 200 consecutive axial slices (20 slices for each of the 10 acquisitions) on the BMMI2 insert (Fig. 1B) using the circular edge technique [19]. TTF values at 50% (*f*_50_, mm^−1^) were used to quantify changes in spatial resolution.

### Detectability index (*d*’)

A non-prewhitening observer model with an eye filter (*d*′_NPWE_) was used to calculate the d’ of two clinical tasks according to the following equation (Eq. 2):

2 $${{d}^{{\prime} }}_{{NPWE}}^{2}=\frac{{\left[\iint {{|W}(u,v)|}^{2}\times {TTF}{\left(u,v\right)}^{2}\times E{\left(u,v\right)}^{2}{dudv}\right]}^{2}}{\iint {{|W}(u,v)|}^{2}\times {TTF}{\left(u,v\right)}^{2}\times {NPS}(u,v)\times {E(u,v)}^{4}{dudv}}$$

where in *u* and *v* are the spatial frequencies in the *x*- and *y*-directions, *E* is the eye filter that models the human visual system sensitivity to different spatial frequencies, and *W(u,v)* is the task function defined according to the following equation (Eq. 3):

3 $$W=|{{FFT}}_{2D}\left\{{h}_{1}\left(x,y\right)-{h}_{0}\left(x,y\right)\right\}|$$

where in FFT is the Fast Fourier Transform, ${h}_{1}\left(x,y\right)$ and ${h}_{0}\left(x,y\right)$ are the object present and object absent hypotheses, respectively [18].

The eye filter was modeled according to the visual response function [20].

Two task functions, each 5 mm in diameter, were defined to represent clinically relevant detection tasks. Their contrasts (290 HU and 201 HU at 40 keV) were chosen to match the reported contrast of non-mucinous and mucinous PMs, respectively, during the portal venous phase [4].

For these two tasks, the signal shape was circular, and the so-called “Gaussian” contrast-profile available on iQMetrix-CT software was used [15]. The following interpretation conditions were considered to calculate *d*′: a display size of 180 mm and a 500-mm viewing distance.

## Statistical analysis

Quantitative variables are presented as means ± standard deviations (SD) [21]. For each metric (noise magnitude, *f*_av_, *f*_50_ and *d*’ values), the relative difference between EID-DECT and SPCCT systems for a specific combination of reconstruction parameter was computed as follows (Eq. 4):

4 $${Mean}\,{difference}\,\left( \% \right)=\frac{{{Metric}}_{{SPCCT}}-{{Metric}}_{{EID}-{DECT}}}{{{Metric}}_{{EID}-{DECT}}}\times 100$$

An average difference of less than ±5% between EID-DECT and SPCCT was considered to be similar.

The optimal parameter combinations with SPCCT were defined as those yielding lower noise magnitude and higher *f*_50_, *f*_av_, and *d*’ values than EID-DECT.

## Patient

To illustrate the image quality of 40-keV VMIs obtained with EID-DECT and with the optimal SPCCT reconstruction parameter combinations, acquisitions were performed on both CT systems in one patient with known non-mucinous PMs during follow-up CT. The same acquisition and reconstruction parameters as those used in the phantom study were employed. However, to more closely replicate clinical conditions, the DoseRight® tube current modulation system was activated on the EID-DECT, and the dose level was therefore adjusted according to the patient’s attenuation. As the tube current modulation system is not available on the SPCCT, the mA settings were adjusted to achieve a CTDI_vol_ as close as possible to that obtained during the CT acquisition on the EID-DECT.

This patient was enrolled as part of a prospective, comparative, monocentric study conducted at a tertiary cardiothoracic university hospital (Hôpital Louis Pradel, Hospices Civils de Lyon, France), with patient enrollment commencing in February 2021 and due to conclude in December 2026. The local ethics committee board had approved the study (Hospices Civils de Lyon, approval number: 2019-A02945–52, SPEQUA study). Two radiologists (S.S.-M. and R.G.) reviewed this patient’s images and assessed the image quality obtained with EID-CT and the various combinations of parameters used with SPCCT by consensus. Visual differences between the images were identified in terms of noise levels and spatial resolution.

## Results

### Noise power spectrum (NPS)

#### Noise magnitude

With SPCCT and both reconstruction kernels, noise magnitude decreased as the iDose^4^ level increased (Table 1). The mean relative decrease in noise magnitude values between successive iDose^4^ levels (i5-i7, i7-i9, and i9-i11) was -28.8 ± 0.5% for STD-B, and -29.4 ± 0.2% for HRB. At each iDose^4^ level, noise magnitude values were higher with HRB than with STD-B, with a mean relative difference of 28.9 ± 1.2% across the four iDose^4^ levels.

**Table 1:** Values for noise magnitude, noise texture (average NPS spatial frequency, *f*_av_), and task-based TTF at 50% (*f*_50_) on BMMI2 inserts obtained for the EID-DECT with Spectral 4 algorithm and SPCCT at the four iDose^4^ levels with the STD-B and HRB kernels

Variables	Algorithms	EID-DECT	SPCCT STD-B	SPCCT HRB
Noise magnitude (HU)	Spectral 4	24.4	-	-
	iDose^4^ 5	-	35.4 (*44.9%*)	46.1 (*88.7%*)
	iDose^4^ 7	-	25.0 (2.4%)	32.4 (*32.7%*)
	iDose^4^ 9	-	17.8 (-27.2%)	22.9 (-6.3%)
	iDose^4^ 11	-	12.7 (-47.8%)	16.2 (-33.6%)
*f*_av_ (mm^-1^)	Spectral 4	0.19	-	-
	iDose^4^ 5	-	0.36 (91.5%)	0.43 (128.7%)
	iDose^4^ 7	-	0.33 (72.9%)	0.40 (114.4%)
	iDose^4^ 9	-	0.28 (47.3%)	0.36 (91.0%)
	iDose^4^ 11	-	0.22 (17.6%)	0.30 (60.1%)
*f*_50_ (mm^-1^)	Spectral 4	0.29	-	-
	iDose^4^ 5	-	0.36 (27.2%)	0.37 (28.6%)
	iDose^4^ 7	-	0.33 (16.2%)	0.31 (8.3%)
	iDose^4^ 9	-	0.30 (5.2%)	0.28 (-0.6%)
	iDose^4^ 11	-	0.30 (3.2%)	0.27 (*-5.1%*)

*EID-DECT* Dual-energy CT equipped with energy-integrating detectors, *SPCCT* Spectral photon-counting CT

Percentage values represent the relative differences between the EID-DECT and SPCCT systems for each reconstruction-parameter combination. The values in italics correspond to those considered not similar and poorer with SPCCT compared with EID-DECT

Compared to EID-DECT, noise magnitude values were higher with SPCCT at level i5 for both kernels. At level i7, the noise magnitude obtained with SPCCT and the STD-B kernel was similar to EID-DECT (25.0 HU *versus* 24.4 HU), whereas higher values were observed with the HRB kernel (32.4 HU). At levels i9 and i11, the noise magnitude was lower than that of EID-DECT for both kernels.

### Noise texture

**Fig. 2:**
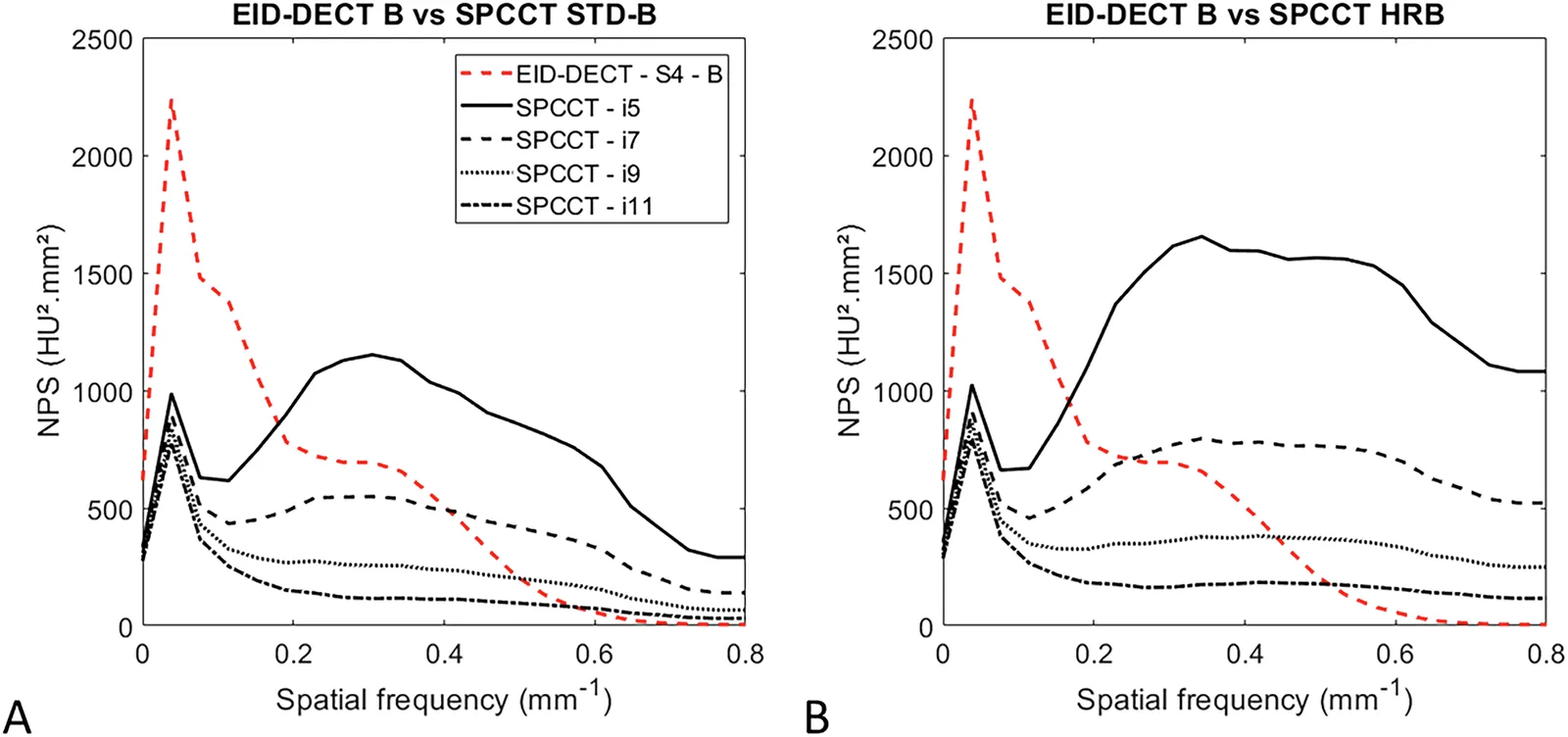
Graphs show NPS1D curves obtained with energy-integrating dual-energy CT (EID-DECT) with Spectral 4 (S4) and kernel B and with SPCCT for STD-B (**A**) and HRB (**B**) reconstruction kernels according to iDose^4^ level

The NPS1D curves obtained with EID-DECT and SPCCT for all parameter combinations are shown in Fig. 2. Two peaks were found on the NPS curves for all EID-DECT and SPCCT images. One peak in the spatial frequencies was found at around 0.04 mm^-1^ and the other one at 0.34 mm^-1^ for EID-DECT, 0.38 ± 0.04 mm^-1^ for SPCCT with HRB, and 0.30 ± 0.05 mm^-1^ with STD-B, across the four iDose^4^ levels.

For SPCCT and both reconstruction kernels, the average NPS spatial frequency (*f*_av_) values decreased as the iDose^4^ level increased (Table 1). For each iDose^4^ level, *f*_av_ values were higher with HRB than with STD-B, and the difference between them increased as the iDose^4^ level increased (from 19.4% at level i5 to 36.2% at level i11).

At all iDose^4^ levels and with both reconstruction kernels, *f*_av_ values were higher with SPCCT than with EID-DECT. Compared to EID-DECT, improvements in *f*_av_ values ranged from 17.6% for level i11 to 91.5% for level i5 with STD-B and from 60.1% to 128.7% for HRB, respectively.

#### Task-based transfer function (TTF)

**Fig. 3:**
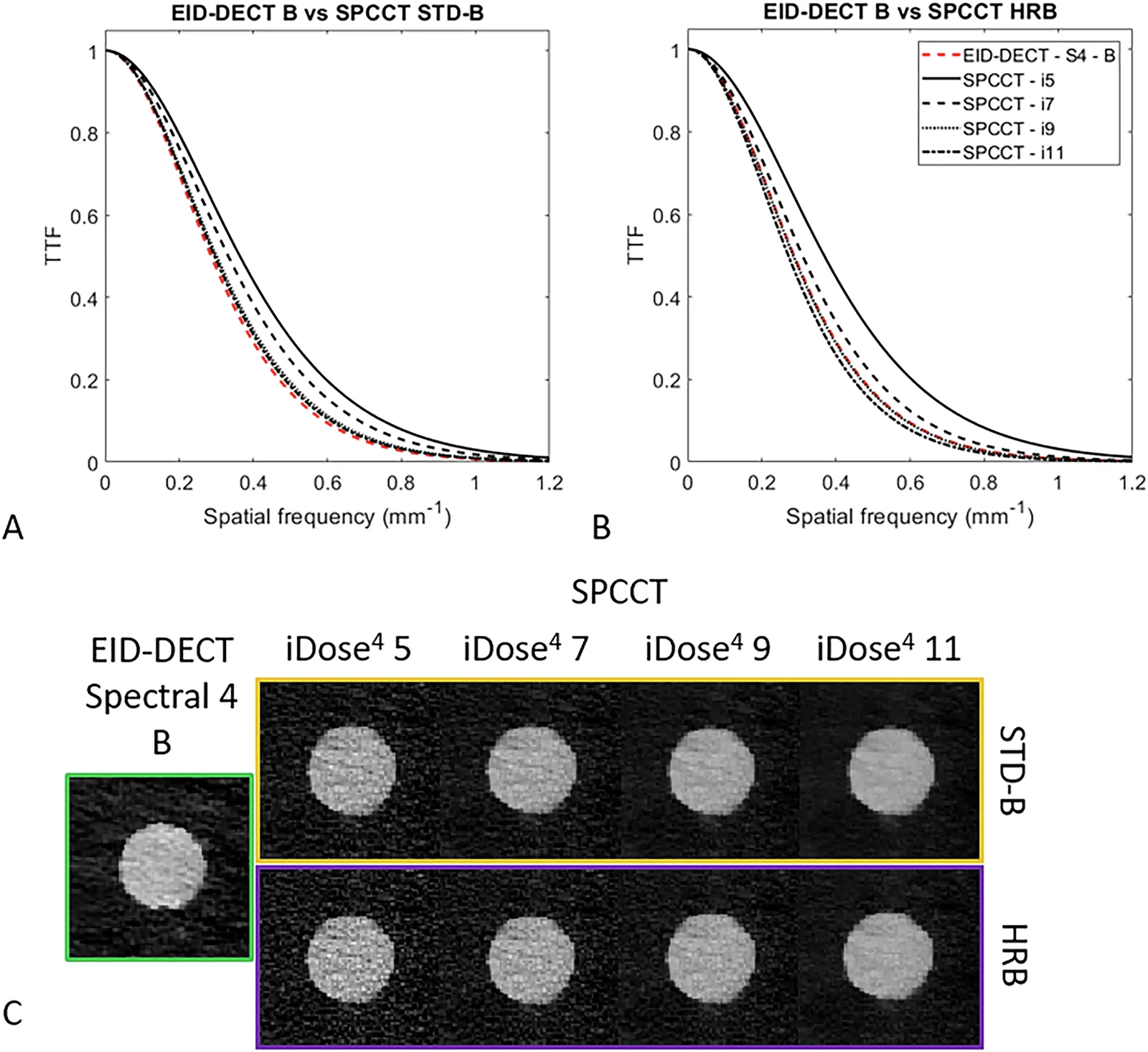
Graphs show task-based transfer function (TTF) curves for the insert of blood-mimicking material plus iodine at 2 mg/mL obtained with energy-integrating dual-energy CT (EID-DECT) with Spectral 4 (S4) and kernel B and SPCCT for STD-B (**A**) and HRB (**B**) reconstruction kernels according to iDose^4^ level. **C** 5.6 × 5.6 cm² region of interest centered on the insert of blood-mimicking material plus iodine at 2 mg/mL (window width: 400 HU and window center: 200 HU)

For SPCCT and both reconstruction kernels, the TTF values at 50% (*f*_50_) decreased as the iDose^4^ level increased (Table 1 and Fig. 3). Similar *f*_50_ values were found with STD-B and HRB at level i5, and *f*_50_ values were higher with STD-B than with HRB for the three other iDose^4^ levels (7.3 ± 1.5%).

For iDose^4^ levels i5 and i7 and both reconstruction kernels, *f*_50_ values were higher with SPCCT than with EID-DECT. At i9 for both kernels, and at i11 for the STD-B kernel, *f*_50_ values were similar to or slightly higher with SPCCT than with EID-DECT. In contrast, at i11, the HRB kernel yielded a slightly lower *f*_50_ value than EID-DECT.

#### Detectability index

**Fig. 4:**
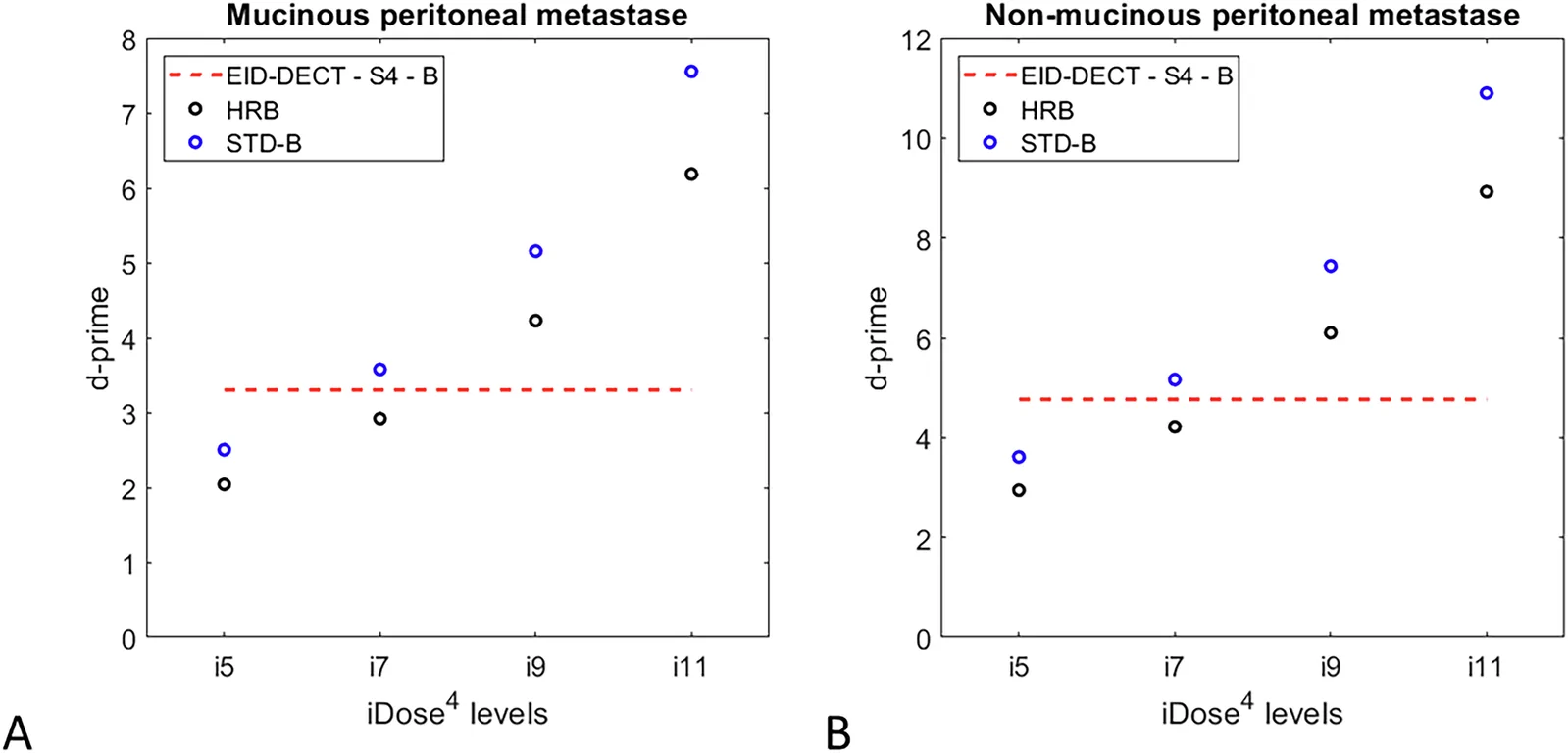
Graphs show *d*’ values of the two simulated lesions obtained with energy-integrating dual-energy CT (EID-DECT) with Spectral 4 (S4) and kernel B and SPCCT for STD-B (**A**) and HRB (**B**) reconstruction kernels according to iDose^4^ level

For SPCCT and both reconstruction kernels, the *d*’ values increased as the iDose^4^ level increased and were higher for non-mucinous PM than mucinous PM at all iDose^4^ levels (Table 2 and Fig. 4). For both reconstruction kernels and both simulated lesions, the mean relative increase in *d*’ values between successive iDose^4^ levels studied was 44.5 ± 1.7% for STD-B, and 44.7 ± 1.3% for HRB.

**Table 2:** Values for the *d*’ of mucinous (mPM) and non-mucinous peritoneal metastases (nmPM) obtained with EID-DECT with the Spectral 4 algorithm and SPCCT for the four iDose^4^ levels with kernels STD-B and HRB

Variables	Algorithms	EID-DECT	SPCCT STD-B	SPCCT HRB
*d*’ mPM	Spectral 4		-	-
	iDose^4^ 5	-	2.51 (*-24.2%*)	2.04 (*-38.2%*)
	iDose^4^ 7	-	3.58 (8.3%)	2.93 (*-11.5%*)
	iDose^4^ 9	-	5.16 (56.0%)	4.23 (27.9%)
	iDose^4^ 11	-	7.56 (128.5%)	6.19 (87.1%)
*d*’ nmPM	Spectral 4		-	-
	iDose^4^ 5	-	3.62 (*-24.2%*)	2.95 (*-38.2%*)
	iDose^4^ 7	-	5.17 (8.3%)	4.22 (*-11.5%*)
	iDose^4^ 9	-	7.44 (56.0%)	6.11 (28.0%)
	iDose^4^ 11	-	10.90 (128.5%)	8.93 (87.1%)

*EID-DECT* Dual-energy CT equipped with energy-integrating detectors, *SPCCT* Spectral photon-counting CT

Percentage values represent the relative differences between the EID-DECT and SPCCT systems for each reconstruction-parameter combination. The values in italics correspond to those considered not similar and poorer with SPCCT compared with EID-DECT

For both simulated lesions, *d*’ values were lower with HRB than with STD-B at each iDose^4^ level, with a mean relative difference of -18.2 ± 0.2% across the four iDose^4^ levels. Compared to EID-DECT, *d*’ values were higher with STD-B for both simulated lesions at all iDose^4^ levels, except for level i5. For HRB and both simulated lesions, the *d*’ values were higher than those obtained with EID-DECT only with levels i9 and i11.

#### Optimal parameter combinations with SPCCT

The best combinations of parameters that met the defined criteria were levels i7, i9, and i11 with STD-B and levels i9 and i11 with HRB. The final choice depended on the objective set by the radiologists and the trade-off between prioritizing an improvement in *f*_av_ and/or *f*_50_ values or an improvement in lesion detectability values.

#### Patient

**Fig. 5:**
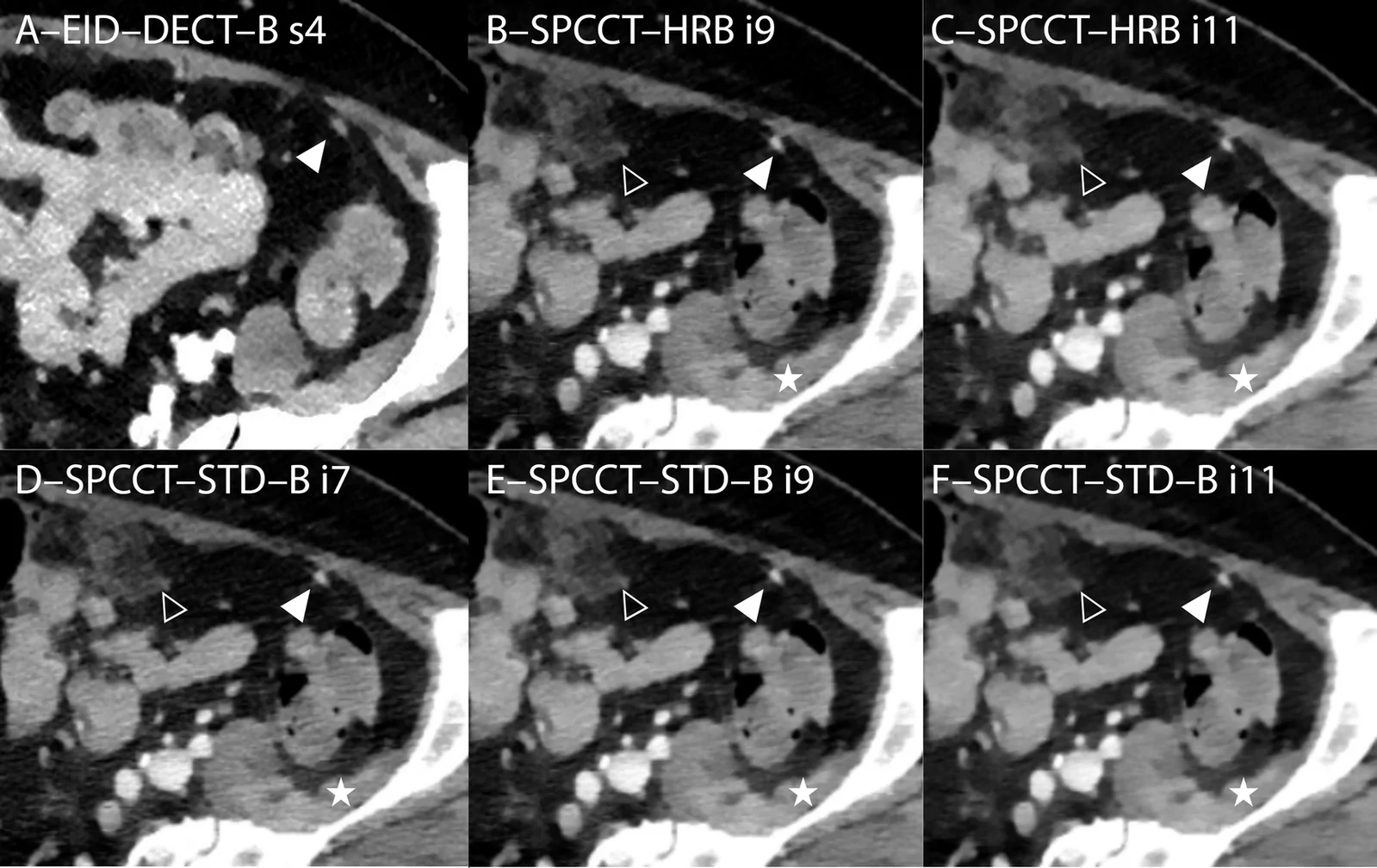
Comparison of abdominal CT images obtained with energy-integrating dual-energy CT (EID-DECT **A**) and SPCCT using different reconstruction parameters (**B**–**F**) on VMIs at 40 keV. The patient was a 59-year-old woman with a body mass index of 24.6 kg/m² presenting with synchronous PMs. Both the EID-DECT and SPCCT acquisitions were performed on the same day, using matched contrast injection parameters: a low-osmolar, nonionic, iodinated contrast agent (Iobitridol, Xenetix 350, Guerbet, 350 mg I/mL) was administered intravenously at a dose of 1.5 mL/kg of body weight at a flow rate of 3 mL/s, with portal venous phase images acquired at a 70-s delay for both scanners. The CTDI_vol_ was 8.2 mGy for EID-DECT and 8.3 mGy for SPCCT. The star indicates the psoas muscle; the full white arrowhead indicates the left parietal peritoneal nodule; and the empty white arrowhead indicates omental fat stranding. Image noise magnitude decreases with increasing iDose⁴ levels, with higher noise observed at i7 compared to levels i9 and i11 for STD-B, as shown in the psoas muscle (star). At the same iDose⁴ level, noise magnitude is slightly higher with the HRB kernel (**B**, **C**) than with the STD-B kernel (**E**, **F**). Increasing iDose⁴ levels are associated with a reduction in spatial resolution, resulting in blurred lesion contours at level i11, as shown with a left parietal peritoneal nodule (full white arrowhead) and omental fat stranding (empty white arrowhead), compared to levels i7 and i9. Overall, compared to EID-DECT, SPCCT images of this patient demonstrated better delineation of muscular fascia. The STD-B kernel at iDose⁴ level 9 provides the best compromise between noise magnitude, noise texture, and spatial resolution

Figure 5 shows the VMI at 40 keV of a patient with PMs obtained with EID-DECT and with the optimal combinations of parameters for SPCCT.

For SPCCT, visual assessment performed by the two radiologists showed that image noise, particularly in the psoas muscle (star), decreased as the iDose⁴ levels increased for both HRB (B and C) and STD-B (D–F) kernels. At the same iDose⁴ level, image noise was slightly higher with the HRB kernel (B and C) than with the STD-B kernel (E and F).

For SPCCT and each kernel, an increase in iDose⁴ level was associated with a decrease in spatial resolution, resulting in blurred lesion margins. This was particularly evident for the left parietal peritoneal nodule (full white arrowhead) and the omental fat streaks (empty white arrowhead) with STD-B at level i11 (F), compared with the images at levels i7 (D) and i9 (E).

## Discussion

In this preliminary phantom and patient study, we investigated the impact of different reconstruction parameters on the quality of 40 keV VMIs obtained with an SPCCT system for detecting PMs. These VMIs were compared with images obtained using the parameters routinely applied in clinical practice on an EID-DECT system [4]. Compared with EID-DECT, SPCCT combined with optimized iterative reconstruction levels and kernels can provide better noise texture, spatial resolution, and lesion detectability.

The NPS results confirmed that noise magnitude and noise texture decreased with increasing iDose⁴ levels for both SPCCT kernels [8, 22]. The noise magnitude and noise texture values were always higher with HRB than with STD-B, regardless of the iDose^4^ level. Two peaks were observed on the NPS1D curves. The low-spatial frequency NPS peaks correspond to known circular artefacts on SPCCT [22] and to non-stationarity at low keV on both CT systems [16]. Regarding the TTF, curves shifted toward lower spatial frequencies as the iDose⁴ levels increased, and *f*_50_ values were higher with STD-B than HRB for all levels except for level i5. These NPS and TTF outcomes translated into slightly noisier but more finely textured images (less pronounced smoothing) with HRB compared with STD-B. Overall, detectability improved for both simulated PM types as the iDose⁴ level increased, with *d*’ values consistently higher for STD-B than for HRB due to the strong influence of noise magnitude on lesion detectability calculation.

The improvement in the metrics studied on a phantom with SPCCT compared to the protocol routinely used in clinical practice with EID-DECT depended on the kernel and iDose^4^ level. With the STD-B kernel, the noise magnitude and *d*’ values for the two simulated PMs were better than, or similar to EID-DECT from levels i7 to i11. For these iDose^4^ levels, the *f*_av_ and *f*_50_ values were also higher, yielding images with finer noise texture, less noise, and slightly better spatial resolution. For the HRB kernel, similar improvements in noise and *d*’ were observed from level i9 onwards, although *f*_50_ values decreased at level i11, indicating a slight loss of spatial resolution. The ideal reconstruction parameters to be used with SPCCT therefore depended on the desired image characteristics. If the main objective was to improve image texture and spatial resolution, STD-B with level i7 offered the best result, although the noise magnitude and detectability remained similar to those of EID-DECT. If the priority was to maximize lesion detectability, STD-B with level i11 was the most appropriate, despite lower *f*_av_ and *f*_50_ values. Overall, STD-B with level i9 offered the best compromise, combining better detectability, finer noise texture, slightly higher spatial resolution, and lower noise than with EID-DECT.

The images obtained on the patient confirmed the phantom findings in a clinical context. SPCCT images demonstrated better delineation of muscular fascia and better depiction of both the parietal peritoneal nodule and omental fat stranding than with EID-DECT, with the STD-B kernel at iDose⁴ level 9 providing the best overall image quality (Fig. 5). These *in vivo* observations, although preliminary, suggest that phantom-derived reconstruction recommendations translate into tangible image quality gains in real patients.

Accurate assessment of PM is critical, both to identify appropriate candidates for cytoreductive surgery at diagnosis, and to reliably estimate the peritoneal cancer index (PCI) before surgery, as incomplete cytoreductive surgery is strongly associated with poorer oncologic outcomes [23]. Detection of PM relies on the right balance between high contrast resolution, ensuring sufficient lesion-to-background differentiation, and high spatial resolution to accurately depict small implants [24]. Adequate contrast between lesions and the adjacent tissues is particularly critical to identifying subtle or infiltrative disease. However, conventional DECT-based approaches remain limited by several technical constraints: the improvement in contrast at low energy levels is accompanied by increased image noise, which may partially offset the diagnostic benefit [8, 9]. Moreover, spatial resolution is inherently restricted by detector design and electronic noise, potentially limiting the detection of millimetric lesions. Concerns regarding radiation dose optimization also persist, as dual-energy acquisitions may involve dose trade-offs depending on the technology and protocols used. By leveraging its advanced capabilities in material decomposition, SPCCT has the potential to enhance the detection of various types of vascularized lesions. This may be especially valuable for poorly enhanced mucinous PM or following neoadjuvant chemotherapy, which are frequently underestimated on conventional CT due to their low attenuation contrast and subtle morphological features [4]. However, these preliminary results are neither a clinical study nor a validation of phantom results, but an *in vivo* clinical demonstration. Our outcomes must now be validated in a cohort of patients for routine abdominal CT.

This study has certain limitations. Acquisitions were performed on a single phantom, which does not account for the wide variability in patient anatomy encountered in abdominal CT examinations. Moreover, the inserts used to simulate lesions do not perfectly replicate the anatomical features of real PMs. The comparison between EID-DECT and SPCCT was based on the reconstruction algorithms currently available for each system for VMIs. Consequently, the improvements observed with SPCCT cannot be attributed solely to PCDs technology, as differences in reconstruction algorithms and their implementation may also have contributed to the image quality and detectability gains. No statistical analysis was performed, as only one calculation of NPS, TTF, and *d*’ was conducted for EID-DECT and only one reconstruction type for SPCCT. Finally, preliminary patient data were presented to support the phantom results; however, this sample is not representative of the full spectrum of patients and lesions encountered in clinical practice. These findings therefore require confirmation in a larger patient cohort.

In conclusion, compared with routine EID-DECT, SPCCT with optimized reconstruction parameters improves image quality and lesion detectability on 40 keV VMIs. Using the STD-B kernel at iDose⁴ level 9 provided the best balance between noise magnitude and texture, spatial resolution, and detectability for simulated PMs. These results indicate that SPCCT may enhance the preoperative assessment of colorectal cancer patients by better visualizing small or low-contrast peritoneal lesions. Validation in larger clinical cohorts is required to confirm these findings and define standardized reconstruction protocols.

## Data Availability

The datasets analyzed during the current study are available from the corresponding author upon reasonable request.

## Abbreviations

CT: Computed tomography
CTDI_vol_: Volume CT dose index
*d*': Detectability index
DECT: Dual-energy CT
EID: Energy-integrating detectors
HU: Hounsfield unit
NPS: Noise power spectrum
PMs: Peritoneal metastases
SPCCT: Spectral photon-counting CT
TTF: Task-based transfer function
VMI: Virtual monoenergetic image
